# Accessory Pathway in Ebstein’s Anomaly: Absence of Evidence Is Not Evidence of Absence

**DOI:** 10.19102/icrm.2022.130304

**Published:** 2022-03-15

**Authors:** Idris Yakut, Funda Özlem Pamuk, Koray Arslan, Ceren Ozdemir, Meryem Kara, Ahmet Korkmaz, Bulent Deveci, Ozcan Ozeke, Serkan Cay, Firat Ozcan, Dursun Aras, Serkan Topaloglu

**Affiliations:** ^1^Department of Cardiology, University of Health Sciences, Ankara City Hospital, Ankara, Turkey

**Keywords:** Ebstein’s anomaly, ECG, electrocardiogram, pre-excitation, RBBB

## Abstract

Electrocardiography has certainly great merit and is almost abnormal in most patients with Ebstein’s anomaly. Incomplete or complete right bundle brunch block (RBBB) is seen in almost half of these patients. The absence of the expected RBBB during sinus rhythm in a patient with Ebstein’s anomaly is a useful clue of pre-excitation in these patients.

A 35-year-old woman with Ebstein’s anomaly was referred for an electrophysiology study of a wide QRS tachycardia (145 bpm) with a right bundle branch block (RBBB) morphology **(Figure 1)**. The electrocardiogram (ECG) after conversion to sinus rhythm by intravenous adenosine showed an inapparent pre-excitation **(Figure 2)**. What do we consider to be the etiology of palpitations based on the ECG? Is the absence of RBBB during sinus rhythm a clue to pre-excitation in Ebstein’s anomaly?

The ECG certainly has great merit and is almost abnormal in most patients with Ebstein’s anomaly.^1,2^ Incomplete or complete RBBB is seen in almost half of these patients, and fragmented triphasic or tetraphasic QRS complexes can be observed due to abnormal conduction through the atrialized right ventricle. The amplitude of the R and R′ in V1 is smaller than that in V5 and V6, contrary to a typical RBBB. Indeed, Marriott introduced the term “rabbit ears” for a double-peaked R-wave in lead V1.^3^ Whereas a “good rabbit” with a taller right peak is typical for an RBBB aberrancy, a “bad rabbit” with a taller left peak suggests a ventricular origin. In other words, the typical RBBB is a triphasic complex best seen in V1 as an rsR′ or rSR′ pattern and in lead I as a qRs or qRS pattern. In the V1 lead, a typical RBBB aberrancy has a small initial r′, as, in RBBB, the high septum is activated primarily from the left septal bundle. Similarly, a typical left bundle branch block has no initial q-wave in lead I and a small r and a rapid S-wave in V1 as it alters the direction of initial septal activation.^4^ On the other hand, ECG in Ebstein’s anomaly of tricuspid valve showed an RBBB pattern with a polyphasic and splintered QRS complex, which can also be considered as a fragmented QRS as a marker of a myocardial scar.^5^ Therefore, the association of giant P-waves with a prolonged P–R interval and an atypical, low-voltage RBBB in the right precordial leads constitutes the hallmark of this entity.^1^ Arrhythmias are another characteristic feature. Pre-excitation and Wolff–Parkinson–White syndrome are more frequently seen in Ebstein’s anomaly than in any other congenital heart disease.^1^ At first glance, the patient seemed to have a sinus rhythm with an inapparent pre-excitation **(Figure 2)**. During the electrophysiological study, a tachycardia was induced, and an atrioventricular accessory pathway (AP) located in the right posterolateral region was detected. After successful ablation, RBBB with a splintering of QRS occurred **(Figure 3)**, which had a similar morphology during tachycardia.

The degree of shortening of the P–R interval and the extent of ventricular pre-excitation depend on several factors. There can be no pre-excitation during sinus rhythm because of slow conduction in the AP or the concealed retrograde penetration of AP. This is suggested by the initial sharp “r” wave in V1. The subsequent conduction along the normal His–Purkinje system would be expected to manifest as an R′ which is abolished by the local pre-excitation by the atrioventricular AP. The proof of this phenomenon was that after ablation in the right posterolateral region (far from the right bundle branch) an RBBB with a splintering of QRS occurs^6,7^ **(Figure 3)**. The RBBB was concealed during sinus rhythm. Thus, in essence, the absence of the expected RBBB during sinus rhythm in a patient with Ebstein’s anomaly is a useful clue of pre-excitation in patients with Ebstein’s anomaly.^5–7^ Moreover, the absence of an RBBB pattern in the surface ECG after ablation should also raise suspicion for the presence of multiple APs.^8^ As per the traditional aphorism, “Absence of evidence is not evidence of absence.”

## Figures and Tables

**Figure 1:**
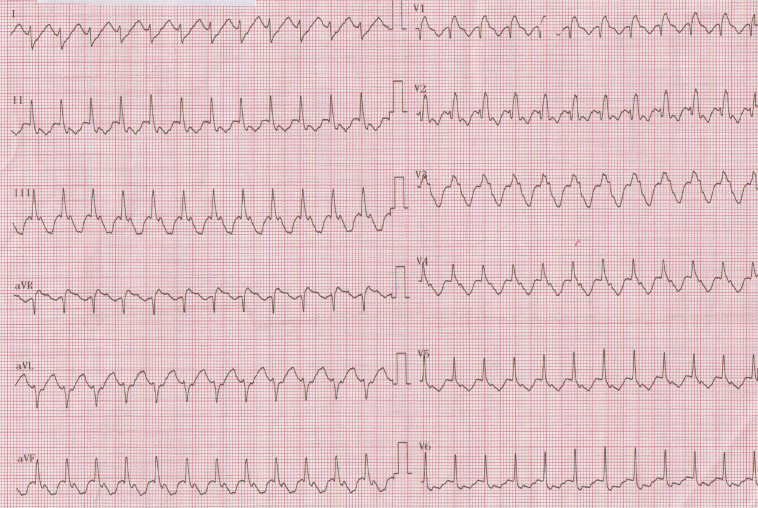
A 12-lead electrocardiogram taken in the emergency department.

**Figure 2:**
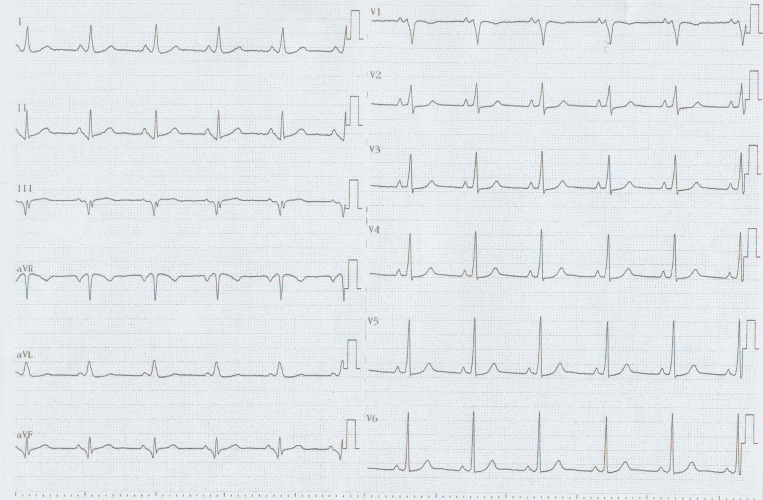
A 12-lead electrocardiogram taken after conversion to sinus rhythm by intravenous adenosine, showing an inapparent pre-excitation.

**Figure 3:**
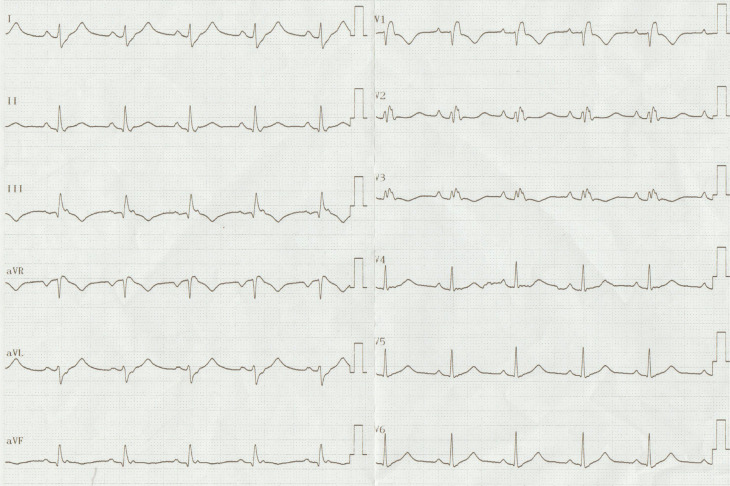
A 12-lead electrocardiogram after successful ablation showing the typical changes, with prolongation of the P–R interval, right bundle branch block, and a somewhat bizarre configuration of the QRS complex.
